# Enhanced diagnostic yield in Meckel-Gruber and Joubert syndrome through exome sequencing supplemented with split-read mapping

**DOI:** 10.1186/s12881-015-0265-z

**Published:** 2016-01-04

**Authors:** Christopher M. Watson, Laura A. Crinnion, Ian R. Berry, Sally M. Harrison, Carolina Lascelles, Agne Antanaviciute, Ruth S. Charlton, Angus Dobbie, Ian M. Carr, David T. Bonthron

**Affiliations:** Yorkshire Regional Genetics Service, St. James’s University Hospital, Leeds, LS9 7TF UK; School of Medicine, University of Leeds, St. James’s University Hospital, Leeds, LS9 7TF UK

**Keywords:** CNV, Exome, Joubert, Meckel-Gruber, Split-read mapping, Whole genome sequencing

## Abstract

**Background:**

The widespread adoption of high-throughput sequencing technologies by genetic diagnostic laboratories has enabled significant expansion of their testing portfolios. Rare autosomal recessive conditions have been a particular focus of many new services. Here we report a cohort of 26 patients referred for genetic analysis of Joubert (JBTS) and Meckel-Gruber (MKS) syndromes, two clinically and genetically heterogeneous neurodevelopmental conditions that define a phenotypic spectrum, with MKS at the severe end.

**Methods:**

Exome sequencing was performed for all cases, using Agilent SureSelect v5 reagents and Illumina paired-end sequencing. For two cases medium-coverage (9×) whole genome sequencing was subsequently undertaken.

**Results:**

Using a standard analysis pipeline for the detection of single nucleotide and small insertion or deletion variants, molecular diagnoses were confirmed in 12 cases (4 %). Seeking to determine whether our cohort harboured pathogenic copy number variants (CNV), in JBTS- or MKS-associated genes, targeted comparative read-depth analysis was performed using FishingCNV. These analyses identified a putative intragenic *AHI1* deletion that included three exons spanning at least 3.4 kb and an intergenic *MPP4* to *TMEM237* deletion that included exons spanning at least 21.5 kb. Whole genome sequencing enabled confirmation of the deletion-containing alleles and precise characterisation of the mutation breakpoints at nucleotide resolution. These data were validated following development of PCR-based assays that could be subsequently used for “cascade” screening and/or prenatal diagnosis.

**Conclusions:**

Our investigations expand the *AHI1* and *TMEM237* mutation spectrum and highlight the importance of performing CNV screening of disease-associated genes. We demonstrate a robust increasingly cost-effective CNV detection workflow that is applicable to all MKS/JBTS referrals.

**Electronic supplementary material:**

The online version of this article (doi:10.1186/s12881-015-0265-z) contains supplementary material, which is available to authorized users.

## Background

In recent years the widespread adoption of next-generation sequencing technologies has led to step-wise improvements in the provision of diagnostic testing services. This was initially a result of an improvement in testing efficiency focusing on single-gene assays, but has more recently been due to ‘panel testing’, whereby numerous genes are analysed concurrently in a single assay. A natural progression of this approach is to perform whole exome sequencing and analyse a “virtual” gene panel, comprising a subset of the sequenced loci. Extremely heterogeneous disorders, overlapping a spectrum of clinical phenotypes, are ideally suited to analysis by these new approaches and have enabled diagnostic laboratories to significantly expand their test portfolios.

We have used virtual exome panels for diagnosis of several heterogeneous autosomal recessive disorders, including primary ciliary dyskinesia (PCD; OMIM: 244400), one of a number of disorders prevalent in our local community [1]. Patients present with recurrent respiratory infections, male infertility and heterotaxy in approximately 50 % of cases. Although cilia are ubiquitous, PCD is caused by defects specifically in the motile cilia that are required for embryonic gastrulation, for clearance of respiratory tract mucosa, and to propel spermatozoa [2]. Standing in contrast are a number of ciliopathies caused by mutations in immotile cilia. These disorders present pleiotropic clinical abnormalities, often of the central nervous system, eye and skeleton, as well as cystic kidney disease. They are relatively common Mendelian conditions with an estimated combined prevalence of 1 in 2000 [3].

Two ciliopathies with overlapping features and considerable underlying genetic heterogeneity are Joubert (JBTS; OMIM: 213300) and Meckel-Gruber (MKS; OMIM: 249000) syndromes, which are currently associated with 31 and 13 genes respectively, ten of which are documented to cause both conditions. JBTS is characterised by neurodevelopmental abnormalities in conjunction with a pathognomonic neuroradiological finding (the molar tooth sign). The latter results from cerebellar vermis hypoplasia or aplasia, elongated superior cerebellar peduncles and a deep interpeduncular fossa. Patients often present with a lack of balance and coordination and a wide spectrum of associated features occur, including postaxial polydactyly, retinal degeneration, cleft lip, seizures, as well as renal and hepatic disease [4]. While some children die in infancy most survive, with variable developmental outcomes. In contrast, MKS is typically a perinatally lethal syndrome characterised by posterior fossa abnormalities (most frequently occipital encephalocele), bilateral enlarged cystic kidneys, postaxial polydactyly and hepatic ductal plate malformation.

Given the overlapping genetic etiologies of these conditions, a pragmatic solution to robust molecular diagnostic investigation is to undertake concurrent mutation analysis of all JBTS/MKS reported genes. Although this strategy is broadly applicable to heterogeneous conditions, individual populations exist in which a more targeted approach is likely to be more cost-effective. One such example is the high prevalence of *MKS1* and *CC2D2A* mutations present in the Finnish population, as a result of founder effects caused by their genetic isolation [5, 6]. Similarly, numerous JBTS patients of French-Canadian origin have been identified with mutations in *TMEM237* or *C5orf42* [7]. Nonetheless, broader routine genetic analysis using NGS gene panels facilitates the establishment of genotype-phenotype correlations in these disorders. The ultimate aim of such analyses is to enable improved variant classification and prognostic assessment. The most compelling reported association is between COACH syndrome (OMIM: 216360), a JBTS-related disorder for which patients have JBTS with liver involvement or coloboma, and mutations in *TMEM67* [8]. In terms of allelic spectrum, missense mutations in *CC2D2A* have been reported to cause JBTS whereas null alleles cause MKS [9]. Such studies are reliant on thorough phenotype data. While this information is often not available to the diagnostic laboratories undertaking the mutation analysis, centralised large-scale resequencing projects are increasingly mandating use of structured phenotype ontologies which will alleviate this problem.

Here we present our experience of using a standard diagnostic pipeline to perform point mutation and small insertion/deletion variant detection in a series of patients referred with either JBTS or MKS. We undertook whole exome sequencing and analysed a virtual panel of JBTS- and MKS-associated genes. We subsequently used these data to undertake exome-based CNV analysis to identify exon deletion and/or duplication variants that are typically smaller than those that can be resolved using standard array CGH platforms. Positive CNV analysis results were validated using whole genome sequencing (WGS) and split read mapping to identify breakpoint sequences at nucleotide resolution.

## Methods

DNA was isolated from blood or fetal tissue of 26 affected individuals using standard salting out or phenol/chloroform extraction protocols. (The developmental age of the individuals ranged from prenatal to 4 years.) All patients were referred to the Yorkshire Regional Genetics Laboratory, Leeds, for diagnostic testing with a clinical diagnosis of either JBTS (9 patients) or MKS (17 patients). The Leeds East Research Ethics Committee granted ethical approval (07/H1306/113) and written informed consent was obtained from the parents or next of kin of all individuals.

### Targeted next-generation sequencing

Diagnostic mutation analysis of JBTS- and MKS-associated genes (Additional file 1: Table S1) was performed using exome-enriched sequence data. Genomic DNA (3 μg) was first sheared into 200–300 bp fragments using a Covaris S2 (Covaris, Inc., Woburn, MA, USA). SureSelect XT reagents (Agilent Technologies, Wokingham, UK) were used to perform end-repair, A-addition and adaptor ligation reactions to generate Illumina-compatible sequencing libraries. Hybridisation capture enrichment of whole genome libraries was performed using the SureSelect v5 all-exon probe set, following manufacturer’s recommendations throughout. Equimolar aliquots of 5 post-enrichment libraries were pooled before sequencing using version 3 TruSeq chemistry on a HiSeq 2500 (Illumina Inc., San Diego, CA, USA). Paired-end 100-bp sequence reads were “demultiplexed” using CASAVA v.1.8.2 and the resulting per-sample FASTQ.gz files were aligned to an indexed reference genome (hg19) using bwa v.0.6.2 (http://bio-bwa.sourceforge.net) [10]. Duplicate reads were removed using Picard v.1.85 (http://picard.sourceforge.net) before indel realignment, base quality score recalibration and variant discovery were performed using the Genome Analysis Toolkit (GATK) v.2.3-4Lite [11]. Identified variants were saved in variant call format (VCF) and then annotated with positional, frequency and functional *in silico* predictions using Alamut Batch standalone v.1.4.0, (database v.2015.04.30) (Interactive Biosoftware, Rouen, France). These programs included SIFT (http://sift.jcvi.org), and AlignGVGD (http://agvgd.iarc.fr/agvgd_input.php). AgileExomeFilter [1] was used to interrogate these data and determine the pathogenicity status of each variant in accordance with the Association for Clinical Genetic Science best practice guidelines [12]. Manual inspection of aligned sequence reads was performed using the Integrative Genome Viewer v.2.3.52 [13]. To determine assay performance, the number of sequence reads mapping to targeted genomic intervals was calculated for each patient using the GATK DepthOfCoverage walker. Exon-based copy number analysis was performed on coordinate-sorted duplicate-cleaned BAM files using FishingCNV v.2.1 [14]. The reference control pool for the copy number analyses comprised 65 patients referred for disorders other than JBTS or MKS. Inter-batch variability was reduced using the FishingCNV principal component option (−pca) for all samples analysed.

### Medium coverage whole genome sequencing

To verify variants identified by FishingCNV, and to validate their intragenic breakpoints, whole genome libraries were generated and sequenced. Approximately 1 μg of DNA was sheared into 200–300-bp fragments using a Covaris S2, and an Illumina-compatible sequencing library generated using NEBNext® Ultra™ reagents (New England Biolabs, Ipswich, MA, USA). The insert size was ~250 bp and the final enrichment PCR comprised six rounds of thermocycling. Each patient library was sequenced on a single lane of a HiSeq 2500 rapid mode flow cell, generating 175-bp sequence reads. Raw data was converted to FASTQ.gz format using CASAVA v.1.8.2. Adaptor sequences were trimmed from the ends of the sequence reads using Cutadapt v.1.1 (https://cutadapt.readthedocs.org) [15] before alignment to the human genome (hg19) using bwa v.0.6.2. Reads that remained unmapped to the reference genome were extracted from the duplicate-cleaned coordinate-sorted BAM files and converted to FASTQ format using bam2fastq v.1.1.0 (http://gsl.hudsonalpha.org/information/software/bam2fastq). For each patient, split read alignments were performed using SplazerS v.1.1 against a FASTA reference sequence that included the FishingCNV-defined CNV (http://www.seqan.de/projects/splazers/) [16]. Breakpoint-spanning reads were identified following interpretation of the alignment CIGAR string. BLAT was then used to determine the genomic coordinates of the 5′ and 3′ fragments (http://genome.ucsc.edu/cgi-bin/hgBlat) [17].

### PCR confirmation assays and Sanger sequencing

PCR amplicons were designed to span SplazerS-identified breakpoints. The primers used to amplify the *TMEM237*/*MPP4* deletion-containing allele were dTGTAAAACGACGGCCAGTACAGGTGGAAGAGCTCGTG (common forward) and dCAGGAAACAGCTATGACCTCTTCAGTATCACCCCAGACA (reverse deletion), which generated a 482-bp product. A second reverse primer (dCAGGAAACAGCTATGACCCCACCACTTTCAGAGGCCAA) was used with the common forward primer to generate a 399-bp PCR product specific for the normal allele. The primers used to amplify the *AHI1* deletion-containing allele were dTGTAAAACGACGGCCAGTTCAAAAGCCCTCTCCTGTAGT (deletion forward) and dCAGGAAACAGCTATGACCATCTTGGGTTTCTGCACACA (common reverse), which generated a 582-bp product. A second forward primer (dTGTAAAACGACGGCCAGTATGTGTCAGGGATCCTCAGG) together with the common reverse primer yielded a smaller 356-bp PCR product specific for the normal allele. Each PCR consisted of 0.5 μl of genomic DNA (250 ng/μl), 11 μl of MegaMix (Microzone Ltd., Haywards Heath, UK), 1 μl of 10 pmol/μl forward primer and 1 μl of 10 pmol/μl reverse primer. Each primer contained a universal tag (underlined) allowing Sanger sequencing according to our standard laboratory workflow. Thermocycling conditions consisted of 5 min at 94 °C then 30 cycles of 94 °C for 30 s, 60 °C for 1 min, and 72 °C for 45 s before a final extension step at 72 °C for 5 min. To facilitate rapid and robust cascade screening, optimal *AHI1* primer concentrations were determined to allow a three-primer multiplex reaction, with PCR products resolved on a 1.5 % tris-borate-EDTA agarose gel.

Sanger sequencing was used to confirm all variants included on clinical reports; manufacturer’s protocols were followed throughout (Applied Biosystems, Paisley, UK). Primer details and thermocycling conditions are available on request. Sequence chromatograms were analysed using Mutation Surveyor v.3.2 (SoftGenetics LLC, State College, PA, USA).

## Results

We have previously implemented a “targeted exome” diagnostic test for primary ciliary dyskinesia [1]. By using the same lab-bench workflow with a modified informatics pipeline, this test has been expanded to include two additional rare recessive conditions, JBTS and MKS. The 34 currently known JBTS and MKS disease-associated genes were included in our analyses; these comprise more than 700 exons and 33 kb of coding sequence (Additional file 1: Table S1). Average read count per patient was 92.4 million reads of which ~9.8 % were identified as PCR duplicates (Additional file 1: Table S2). This is slightly higher than our typical duplicate rate and may reflect the limited quantity and quality of the available DNA samples. The percentage of reads mapping to exome-located nucleotides (~59 %) is consistent with previous reports [18] and corresponds to ~48 million reads per exome. Approximately 86 % of target nucleotides per sample (coding bases plus 20-bp of flanking intron) were sequenced by at least 30 reads (Additional file 1: Table S3).

Variant interrogation was restricted to the 34 known disease-associated genes. This reduced the average total variant count from 33,766 to 70 per patient (Additional file 1: Table S4). By excluding common variants (minor allele frequency 0.05) the burden of manual interpretation was reduced to approximately eight variants per patient. The disease-associated pathogenicity status of each of these was classified according to the Association for Clinical Genetic Science best practice guidelines [12]. In this way a confirmatory molecular diagnosis was achieved in 12 out of 26 cases (46 %) (Table 1). The mutation spectrum was largest for *TMEM67*, which harboured seven different pathogenic mutations in our cohort. The most frequently identified pathogenic mutation was *TCTN2* c.1506-2A > G (p.?) [GenBank:NM_024809.4], which was detected seven times.

**Table 1:** Pathogenic variants identified following routine diagnostic testing

Sample number	Gene	Transcript	Allele 1	Allele 2	Reference(s)
1	*AHI1*	NM_001134830.1	c.1983del (p.Trp662Glyfs*24)	No mutation detected	
2	*AHI1*	NM_001134830.1	c.2495del (p.Leu832*)	c.2495del (p.Leu832*)	[24, 25]
3	*CC2D2A*	NM_001080522.2	c.2803C > T (p.Arg935*)	c.3774dup (p.Glu1259*)	
4	*CC2D2A*	NM_001080522.2	c.2875del (p.Glu959Asnfs*3)	c.2875del (p.Glu959Asnfs*3)	
5	*CEP290*	NM_025114.3	c.1975A > T (p.Lys659*)	c.5668G > T (p.Gly1890*)	[26–28]
6	*MKS1*	NM_017777.3	c.262-2A > G (p.?)	No mutation detected	
7	*TCTN2*	NM_024809.4	c.1506-2A > G (p.?)	No mutation detected	[29]
8	*TCTN2*	NM_024809.4	c.1506-2A > G (p.?)	c.1506-2A > G (p.?)	[29]
9	*TCTN2*	NM_024809.4	c.1506-2A > G (p.?)	c.1506-2A > G (p.?)	[29]
10	*TCTN2*	NM_024809.4	c.1506-2A > G (p.?)	c.1506-2A > G (p.?)	[29]
11	*TMEM67*	NM_153704.5	c.415_416del (p.Asp139Hisfs*2)	c.415_416del (p.Asp139Hisfs*2)	
12	*TMEM67*	NM_153704.5	c.514C > T (p.Arg172*)	c.622A > T (p.Arg208*)	[30, 31]
13	*TMEM67*	NM_153704.5	c.579_580del (p.Gly195Ilefs*13)	c.579_580del (p.Gly195Ilefs*13)	[32]
14	*TMEM67*	NM_153704.5	c.1319G > A (p.Arg440Gln)	c.1319G > A (p.Arg440Gln)	[31–34]
15	*TMEM67*	NM_153704.5	c.1960 + 1G > A (p.?)	c.1046 T > C (p.Leu349Ser)	[32, 33, 35]
16	*TMEM237*	NM_001044385.2	c.709del (p.Ala237Leufs*10)	No mutation detected	

In patients, the diagnosis of JBTS or MKS could not be confirmed, either (a) because the identified variant(s) was classified as probably benign, or a variant of uncertain significance or (b) because the patient was a carrier of only a single *bona fide* pathogenic mutation (four individuals). We performed exome-based copy number analysis of the JBTS- and MKS-associated disease genes for all patients in the cohort (data not shown). Heterozygous multi-exon deletions were identified in two separate patients. Both of these subjects were also carriers of pathogenic single-nucleotide deletions in the corresponding gene. For patient 1 the deletion reported by FishingCNV spanned at least 3.4 kb and included *AHI1* exons 14–16 [GenBank:NM_001134830.1] (Additional file 2: Figure S1). For patient 16, the detected deletion extended from *TMEM237* exon 1a [GenBank:NM_152388.3] to *MPP4* exon 14 [GenBank:NM_003066.2], spanning at least 21.5 kb and 11 exons. For both variants, the maximal extent of the deleted sequence was defined by the proximity of captured flanking exons; this refined the maximal extent of each deletion to 9.9 kb and 25.5 kb, respectively.

**Fig. 1:**
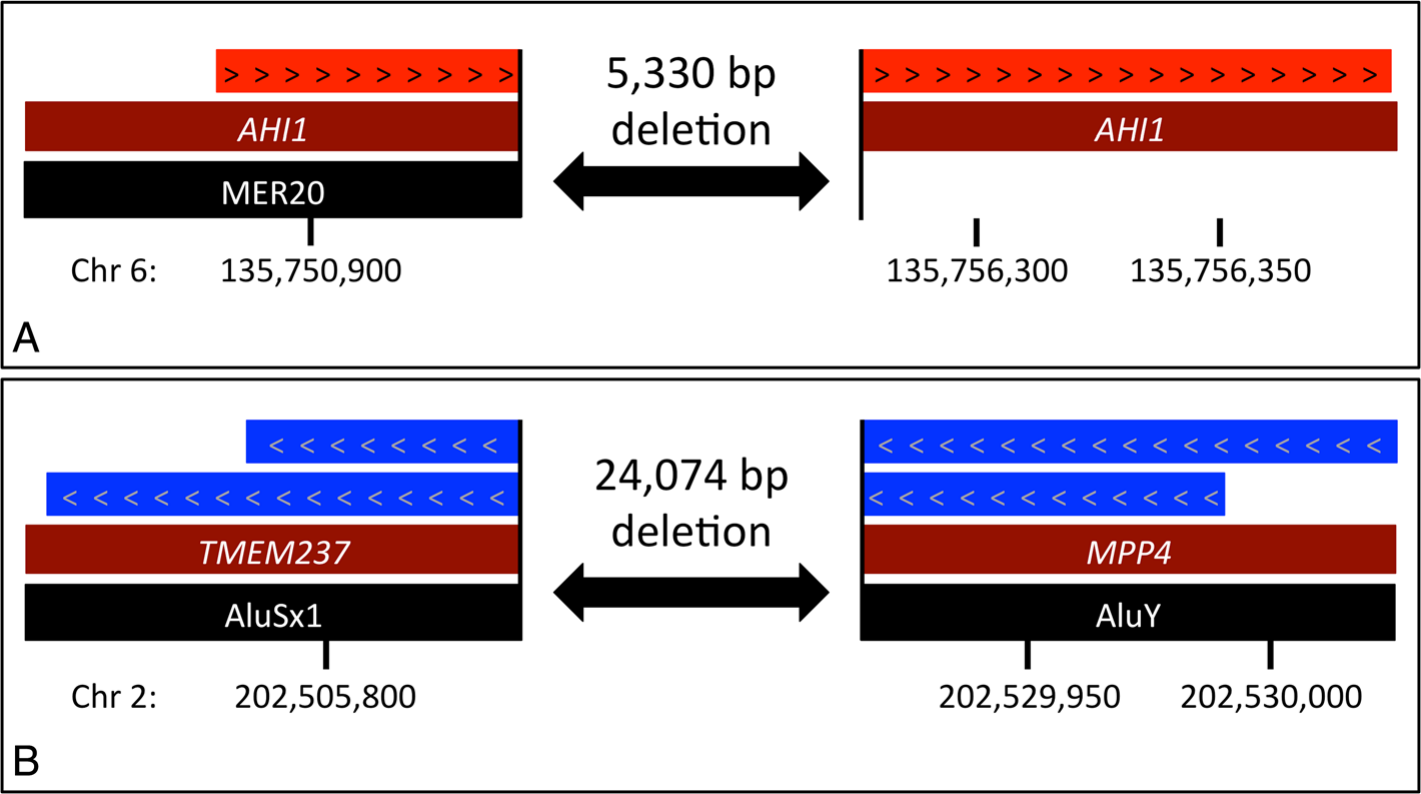
A schematic representation of SplazerS-identified breakpoint-spanning reads for (**a**) the intragenic *AHI1* deletion and (**b**) the intergenic *TMEM237* to *MPP4* deletion. Red and blue arrowed boxes denote reads mapping to the (+) and (−) strands respectively. Black tracks contain the names of RepeatMasker-identified sequence elements. Genomic coordinates are for build hg19. Chromosome

Retrospective inspection of aligned sequence reads within the putative disease-containing loci did not reveal any homozygous non-reference (or heterozygous) variants to support (or oppose) the existence of the deleted sequences. Inspecting the alignment BAM files in the Integrated Genome Viewer did however reveal a halving of relative read depth at both deletion-containing loci. To validate our findings and delineate the intragenic breakpoints, we performed medium-coverage (9×) WGS using a longer, 175-bp sequencing read. To reduce adaptor read-through, the library insert, size was increased from ~200 bp to ~250 bp. Despite this, ~20 % adaptor-trimmed reads were present in the sequenced dataset (Table 2). Unmapped reads were recovered from the alignment BAM file and a split-read mapping algorithm was used to search for reads spanning the deletion breakpoint. For the *AHI* variant, a single such read was identified, indicating a deletion of 5,330 nucleotides, while for the *TMEM237*/*MPP4* variant two reads were identified, suggesting a deletion of 24,074 nucleotides. The number of aligned nucleotides on either side of the breakpoint and their genomic coordinates, identified using BLAT, are listed in Table 3. The positions and orientations of these breakpoint-spanning reads with respect to the human reference sequence and UCSC RepeatMasker track are shown in Fig. 1. The centromeric end of the *AHI1* variant intersects a *MER20* DNA repeat and the *TMEM237*/*MPP4* variant intersects two low complexity *Alu* repeat elements.

**Table 2:** Summary metrics for medium coverage whole genome sequencing data

Sample number	Read type	Raw read count	Trimmed reads (%)	Reads identified as duplicates (%)	Mapped reads^a^	Unmapped reads
1	175-bp SR	166055253	18.1	4.20	141464444	18391865
16	175-bp SR	141082846	21.0	4.32	117693041	18076624

*SR* single read

^a^Following duplicate removal

**Table 3:** Characteristics of SplazerS-mapped breakpoint spanning reads

Sample number	Read ID	5’ match (nt)	3’ match (nt)	Trimmed read length (nt)	Strand	Chr	5’ match start	5’ match stop	3’ match start	3’ match stop
1	1:1207:14146:94852	64	111	175	+	6	135750881	135750944	135756275	135756385
16	2:2114:3289:41334	112	58	170	-	2	202529915	202530026	202505783	202505840
16	2:2115:10757:93844	76	99	175	-	2	202529915	202529990	202505742	202505840

Genomic coordinates are provided for hg19 with respect to the strand from which the read was sequenced

*nt* nucleotides, *Chr* chromosome

**Fig. 2:**
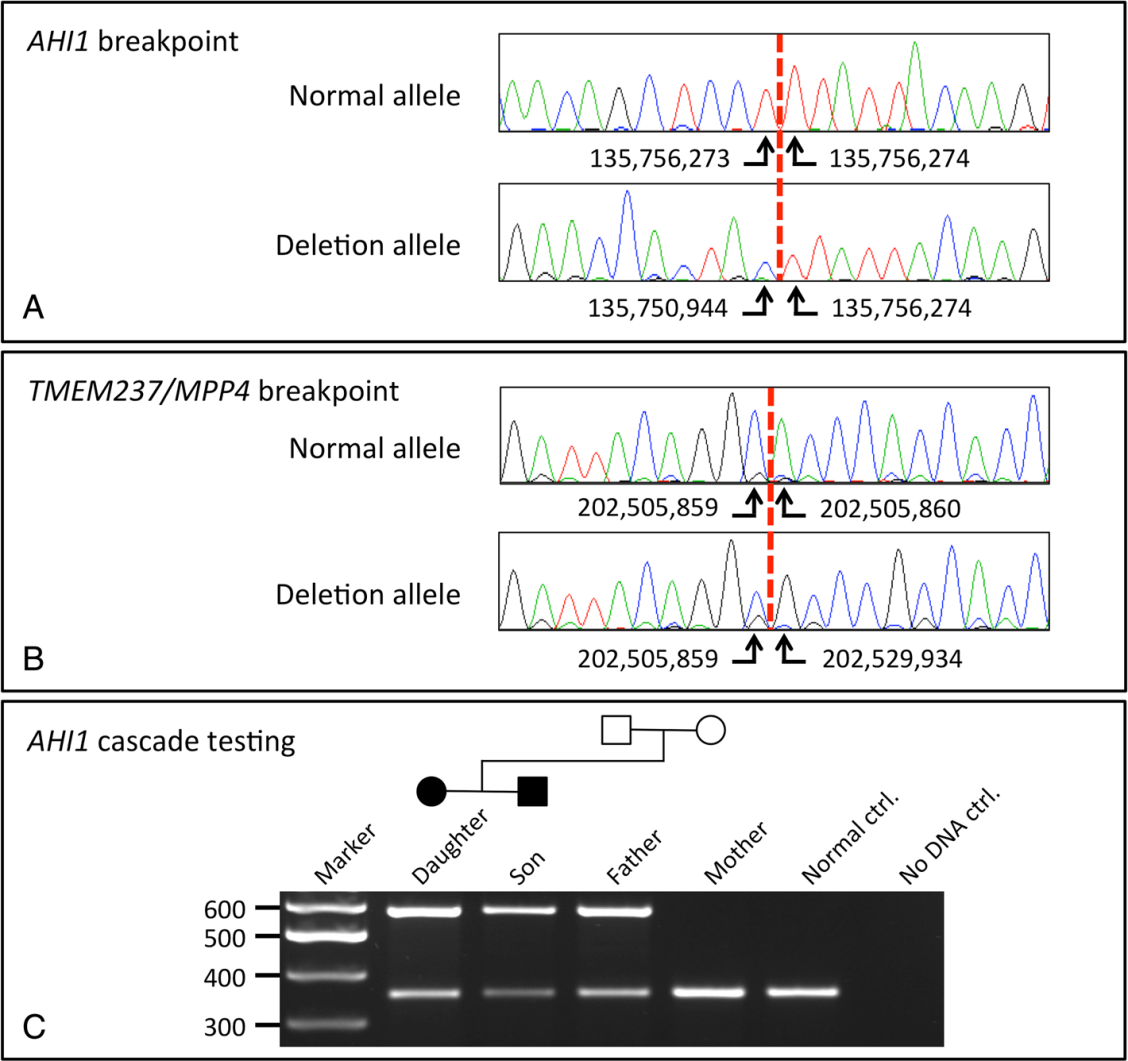
Sanger sequencing chromatograms verifying the deletion breakpoints for (**a**) the intragenic *AHI1* deletion and (**b**) the deletion spanning *TMEM237* to *MPP4*. **c** A diagnostic multiplex PCR assay showing amplification products for normal (356-bp) and deletion-containing (582-bp) *AHI1* alleles. Both affected siblings are heterozygous for the deletion-containing allele, which was inherited from their father

Sanger sequencing of breakpoint-spanning PCR products confirmed the identity of both amplicons and that the breakpoints were clean deletions with no inserted nucleotides (Fig. 2a and b). To genotype the multi-exon *AHI1* deletion variant in the extended family a multiplex PCR was used which incorporated both the normal- and deletion-specific forward primers in conjunction with a common reverse primer (Fig. 2c). In heterozygous mutation carriers, a larger mutation-specific PCR product is visible in addition to the normal band. The assay confirmed that the proband inherited the deletion-containing allele from her father and that her affected brother was also heterozygous for the mutation. In conjunction with the segregation of the pathogenic *AHI1* single-base deletion, c.1983del (p.Trp662Glyfs*24), this confirmed that the two variants were present in *trans* in both affected siblings.

## Discussion

NGS workflows suitable for detecting single nucleotide and small insertion/deletion variants are now well established throughout the genetic diagnostic community. Many assays initially used long-range PCR as the target enrichment method [19], but owing to the finite number of long-range PCR primers that can be sustainably handled, enrichment is now more typically performed using hybridisation capture. Furthermore, the robustness and quality assurance provided by a pre-designed “off-the-shelf” reagent, as well as the potential to target much larger genomic regions, has added to the increased popularity of this methodology. For staff undertaking NGS library production, a single laboratory method streamlines the workflow and increases productivity. For analysis, establishing a new diagnostic test can be as straightforward as adjusting the genomic coordinates of the targeted regions. These factors are enabling diagnostic laboratories to maintain pace with the emergence of newly reported gene associations. In our centre, we now routinely undertake targeted diagnostic exome analysis of JBTS- and MKS-associated genes. We were able to confirm diagnoses in 14 of the first 26 cases, a diagnostic yield of 54 %. This may increase in light of new evidence, as it is possible that variants deemed to be of uncertain clinical significance, may be re-classified as pathogenic, disease-causing, mutations. Alternatively, the “missing” disease-causing mutations may be located in genomic regions that were not targeted either for sequencing (e.g. promoters or introns) or analysis (genes yet to be recognised as disease-associated). For example, common biological mechanisms involving ciliary structure and function may imply genetic relatedness between JBTS/MKS and the wider spectrum of “ciliopathies”, in which developmental effects of defective ciliary function are manifested. Our strategy of analysing a full exome, rather than a gene set targeting only OMIM genes (a “clinical exome”) will allow us to re-analyse quickly, as new disease-associated genes continue to be reported. It is likely that this will be driven by multi-centre consortia capable of amassing many hundreds of similarly affected patients as has been recently reported by Bachmann-Gagescu et al. [20]. The diagnostic yield in their study was comparable with our own. Despite this the spectrum of identified pathogenic mutations was more diverse, comprising mutations in 23 of the 27 genes they analysed. The considerable number of cases of South Asian ethnicity (13 patients), for whom consanguineous marriage is customary, may account for the relative homogeneity of pathogenic mutations identified in our cohort. That the *TCTN2* mutation c.1506-2A > G was identified in four individuals, all of whom were South Asian, suggests this variant may be a founder mutation in this population. Seventeen cases (65 %) were referred for testing with a clinical diagnosis of MKS, so it is unsurprising that an MKS locus accounts for the greatest number of different pathogenic variants in our cohort. Given the accelerating rate of new gene discovery, the ability to reanalyse existing datasets is likely to be of particular clinical utility [21].

Comparative read-depth analysis enabled the identification of two heterozygous deletion variants in *trans* with pathogenic point mutations. It is worth noting that the use of whole exome sequencing allowed us to better define the maximal extent of the intergenic *TMEM237*/*MPP4* deletion than would have been possible had we used a reagent targeting only known JBTS/MKS genes. (If limited sequencing capacity precludes the use of routine exome sequencing, one alternative approach might be to add capture probes for the exons of genes flanking the targeted genes.) Although multiplex-ligation probe (MLPA) assays are widely regarded as the “gold-standard” technique for detecting exonic deletions and duplications, the production of validated reagents cannot keep pace with ever expanding targeted gene panels. In contrast, our CNV analyses did not require additional laboratory reagents or staff costs.

While it is conceptually and practically straightforward to identify homozygous deletions, due to the absence of sequence reads at normally well-captured genomic loci, automated identification of heterozygous deletions and duplications is more challenging and the sensitivity of CNV detection remains poorly defined. Limitations probably include reduced sensitivity of detection of CNVs encompassing GC-rich regions. Such regions, which often include a gene’s first exon, are consistently reported to be poorly captured using hybridisation enrichment technologies [22]. Consequently, custom capture reagent designs, targeting fewer loci than an exome, may overcome problems associated with regions of repeatedly poor sequence coverage by allowing an increase in capture probe density in specific underrepresented regions. An important consideration when performing relative read-depth analyses is the origin of the control dataset, specifically whether it is derived from inter-batch or intra-batch samples. In targeted exome analyses, available sequencer capacity probably limits the ability of most diagnostic laboratories to pool enough samples in one experiment for robust intra-batch normalisation. This approach may therefore be more amenable to the analysis of smaller targeted panels, in which more NGS libraries can be pooled. Regardless of the method used to define the control cohort, knowledge of a disorder’s mutation spectrum is of paramount importance. Including in a reference control pool cases for which there is a high prior probability of a CNV (or including family members) could dilute the effect of a true deviation in relative read-depth.

The ability to detect CNVs in hybridisation enrichment experiments is influenced by gene size and structure. A deletion of large genomic extent could be missed if exons are sparsely distributed, reducing the number of available data-points. Similar considerations apply when trying to define the boundaries of a putative CNV. Consequently, for genes within which CNVs account for a significant component of the mutation spectrum, it may prove beneficial to include additional capture probes within surrounding introns. Although our investigations have revealed two cases in whom dosage variants account for the pathogenic disease allele, we have identified too few events to ascribe a precise sensitivity and specificity to this workflow. Future efforts in this area are likely to be hindered, in the short-term, by the paucity of identified pathogenic copy-number alleles. As such, without performing whole genome sequencing of the entire cohort, it will not be possible to completely exclude pathogenic dosage mutations in JBTS or MKS disease associated genes in the reported patients.

We confirmed candidate CNVs using medium coverage (9×) WGS, an increasingly cost-effective solution which enabled nucleotide-resolution breakpoint detection to be delivered in a regional diagnostic laboratory. We used an unsupported 175-bp sequencing protocol, but the recent release of 2 × 250-bp kits should further improve the power of this approach to CNV validation. The length of read available for alignment is especially important when performing split-read alignments, as the breakpoint will not in general be centred within the read. The sequence context surrounding the breakpoint will also have a direct bearing on its ability to be detected. Both breakpoints reported here were located within elements identified by RepeatMasker. Low complexity sequences are difficult to align to a reference sequence using short read technology. Although we were able to implement customised sequencer run configurations, these will not be available to all diagnostic laboratories. For these centres, the identification of discordant read pairs (for which the read mapping positions are at variance with the expected library insert size) may offer an alternative route to validating CNVs.

Defining CNVs at single-nucleotide resolution permits inexpensive PCR-based genotyping assays to be deployed for testing the extended family, as demonstrated above for case 1.

## Conclusions

The CNVs identified here have not previously been reported in the literature and therefore expand the JBTS mutation spectrum. With ever-improving mutation detection techniques, it is likely that dosage variants will be more frequently identified and characterised at nucleotide resolution. The CNVs we describe are smaller than those typically detected by diagnostic array-CGH or by our standard genome-wide copy number sequencing (CNVseq) approach [23]. Although the sensitivity gap between front-line aCGH/CNVseq technologies and the limits of detection using the comparative read-depth approach are ill-defined, they will probably be bridged as the cost of WGS falls in future years. In the interim, our present report, emphasizes the additional diagnostic yield that can be obtained at little cost, using modifications of current analytical approaches.

### Availability of supporting data

All reported variants have been uploaded to an LOVD database accessible at http://databases.lovd.nl/shared.

## Additional files

Additional file 1: Table S1. Joubert and Meckel-Gruber syndrome genes included in the targeted exome analysis. **Table S2.** Summary performance metrics for each sample analysed. **Table S3.** The percentage of target nucleotides for each gene with a read depth ≥30. **Table S4.** Summary variant counts following data processing using the SNV/small indel detection pipeline. (DOCX 190 kb) Additional file 2: Figure S1. Schematic representation of the FishingCNV-defined deletions showing the minimum (red) and maximum (green) possible boundaries of the deletion breakpoints for (A) the intragenic *AHI1* deletion and (B) the *TMEM237* to *MPP4* deletion. Exons are displayed in blue and numbering is in accordance with transcripts [GenBank:NM_001134830.1] (*AHI1*), [GenBank:NM_001044385.2] and [GenBank:NM_152388.3] (*TMEM237*) and [GenBank:NM_033066.2] (*MPP4*). (TIF 19237 kb)

## Abbreviations

CNV: copy number variant
CNVseq: copy number variation sequencing
JBTS: Joubert
MKS: Meckel-Gruber
MLPA: multiplex-ligation probe
PCD: primary ciliary dyskinesia
VCF: variant call format
WGS: whole genome sequencing
