# Supplementary material for: Enhanced diagnostic yield in Meckel-Gruber and Joubert syndrome through exome sequencing supplemented with split-read mapping
Source: BMC Med Genet. 2016 Jan 4;17:1. doi: 10.1186/s12881-015-0265-z (PMC4700600; doi:10.1186/s12881-015-0265-z)
Supplement: Additional file 1: Table S1. — Joubert and Meckel-Gruber syndrome genes included in the targeted exome analysis. Table S2. Summary performance metrics for each sample analysed. Table S3. The percentage of target nucleotides for each gene with a read depth ≥30. Table S4. Summary variant counts following data processing using the SNV/small indel detection pipeline. (DOCX 190 kb) [file 12881_2015_265_MOESM1_ESM.docx]

**Supplementary Table 1: Joubert and Meckel-Gruber syndrome genes included in the targeted exome analysis**

| **Gene** | **OMIM ID(s)** | **OMIM phenotype(s)** | **Location** | **Chr** | **Start*** | **Stop*** | **Strand** | **Transcript** | **Exons** | **Amino acids** |
| --- | --- | --- | --- | --- | --- | --- | --- | --- | --- | --- |
| *AHI1* | 608629 | JBTS3 | 6q23.3 | 6 | 135,818,903 | 135,605,110 | - | NM_001134830.1 | 27 | 1,196 |
| *ARL13B* | 612291 | JBTS8 | 3q11.1 | 3 | 93,698,983 | 93,774,522 | + | NM_001174150.1 | 10 | 428 |
| *B9D1* | 614209 | MKS9 ¶ | 17p11.2 | 17 | 19,266,046 | 19,246,483 | - | NM_001243473.1 | 6 | 257 |
| *B9D2* | 614175 | MKS10 ¶ | 19q13.2 | 19 | 41,870,078 | 41,860,322 | - | NM_030578.3 | 4 | 175 |
| *C2CD3* | 615948 | ¶ | 11q13.4 | 11 | 73,882,064 | 73,745,477 | - | NM_015531.4 | 31 | 1,963 |
| *C5orf42* | 614615 | JBTS17 | 5p13.2 | 5 | 37,249,530 | 37,106,330 | - | NM_023073.3 | 52 | 3,197 |
| *CC2D2A* | 612285 / 612284 | JBTS9 / MKS6 | 4p15.32 | 4 | 15,471,489 | 15,603,180 | + | NM_001080522.2 | 38 | 1,620 |
| *CEP41* | 614464 | JBTS15 | 7q32.2 | 7 | 130,081,051 | 130,033,612 | - | NM_018718.2 | 11 | 373 |
| *CEP104* |  | ¶ | 1p36.32 | 1 | 3,773,797 | 3,728,645 | - | NM_014704.3 | 22 | 925 |
| *CEP290* | 610188 / 611134 | JBTS5 / MKS4 | 12q21.32 | 12 | 88,535,993 | 88,442,790 | - | NM_025114.3 | 54 | 2,479 |
| *CSPP1* | 615636 | JBTS21 | 8q13.1-q13.2 | 8 | 67,976,603 | 68,108,849 | + | NM_024790.6 | 29 | 1,221 |
| *IFT172* | 615630 | ¶ | 2p23.3 | 2 | 27,712,678 | 27,667,240 | - | NM_015662.2 | 48 | 1,749 |
| *INPP5E* | 213300 | JBTS1 | 9q34.3 | 9 | 139,334,256 | 139,323,067 | - | NM_019892.4 | 10 | 644 |
| *KIAA0586* | 616490 | JBTS23 | 14q23.1 | 14 | 58,894,710 | 59,015,549 | + | NM_001244189.1 | 34 | 1,644 |
| *KIF7* | 200990 | JBTS12 | 15q26.1 | 15 | 90,198,682 | 90,171,201 | - | NM_198525.2 | 19 | 1,343 |
| *KIF14* | 616258 | MKS12 | 1q32.1 | 1 | 200,589,862 | 200,520,625 | - | NM_014875.2 | 30 | 1,648 |
| *MKS1* | 249000 | MKS1 ¶ | 17q22 | 17 | 56,296,666 | 56,282,797 | - | NM_017777.3 | 18 | 559 |
| *NPHP1* | 609583 | JBTS4 | 2q13 | 2 | 110,962,639 | 110,880,914 | - | NM_000272.3 | 20 | 733 |
| *NPHP3* | 267010 | MKS7 | 3q22.1 | 3 | 132,441,303 | 132,399,453 | - | NM_153240.4 | 27 | 1,330 |
| *OFD1* | 300804 | JBTS10 | Xp22.2 | X | 13,752,832 | 13,787,480 | + | NM_003611.2 | 23 | 1,012 |
| *PDE6D* | 615665 | JBTS22 | 2q37.1 | 2 | 232,646,037 | 232,597,135 | - | NM_002601.2 | 5 | 150 |
| *PIBF1* |  | ¶ | 13q21.33 | 13 | 73,356,230 | 73,590,591 | + | NM_006346.2 | 18 | 757 |
| *RPGRIP1L* | 611560 / 611561 | JBTS7 / MKS5 | 16q12.2 | 16 | 53,737,771 | 53,633,818 | - | NM_015272.2 | 27 | 1,315 |
| *TCTN1* | 614173 | JBTS13 | 12q24.11 | 12 | 111,051,832 | 111,086,935 | + | NM_001082538.2 | 15 | 592 |
| *TCTN2* | 613885 | MKS8 ¶ | 12q24.31 | 12 | 124,155,660 | 124,192,950 | + | NM_024809.4 | 18 | 697 |
| *TCTN3* | 614815 | JBTS18 | 10q24.1 | 10 | 97,453,900 | 97,423,153 | - | NM_015631.5 | 14 | 607 |
| *TMEM67* | 610688 / 607361 | JBTS6 / MKS3 | 8q22.1 | 8 | 94,767,072 | 94,830,347 | + | NM_153704.5 | 28 | 995 |
| *TMEM107* |  | § | 17p13.1 | 17 | 8,079,714 | 8,076,297 | - | NM_032354.3 | 5 | 146 |
| *TMEM138* | 614465 | JBTS16 | 11q12.2 | 11 | 61,129,473 | 61,136,975 | + | NM_016464.4 | 5 | 162 |
| *TMEM216* | 608091 / 603194 | JBTS2 / MKS2 | 11q12.2 | 11 | 61,159,832 | 61,166,335 | + | NM_001173991.2 | 5 | 148 |
| *TMEM231* | 614970 | JBTS20 / MKS11 | 16q23.1 | 16 | 75,590,184 | 75,572,015 | - | NM_001077416.1 | 6 | 345 |
| *TMEM237* | 614424 | JBTS14 | 2q33.1 | 2 | 202,508,252 | 202,484,907 | - | NM_001044385.2 | 13 | 408 |
| *TTC21B* | 613820 | JBTS11 | 2q24.3 | 2 | 166,810,348 | 166,729,872 | - | NM_024753.4 | 29 | 1,316 |
| *ZNF423* | 614844 | JBTS19 | 16q12.1 | 16 | 49,856,649 | 49,524,515 | - | NM_015069.3 | 8 | 1,284 |

Data reproduced from OMIM (http://www.omim.org), last accessed 27^th^ May 2015. JBTS: Joubert syndrome; MKS: Meckel-Gruber syndrome. ¶; Associated with JBTS but no assigned OMIM phenotype. §; Associated with MKS but no assigned OMIM phenotype. Chr: Chromosome. *RefSeq gene coordinates for build hg19.

**Supplementary Table 2: Summary performance metrics for each sample analysed**

| **Sample**  **number** | **Raw read count** | **Reads identified as duplicates (%)** | **Median insert size (bp)** | **Total number of mapped reads*** | **Number of reads mapped to coding exons*** | **Reads**  **on target (%)** |
| --- | --- | --- | --- | --- | --- | --- |
| 1 | 100,476,702 | 9.62 | 189 | 89,468,027 | 50,668,277 | 56.63 |
| 2 | 75,415,814 | 5.97 | 199 | 69,976,071 | 40,250,552 | 57.52 |
| 3 | 104,614,700 | 10.79 | 183 | 91,817,162 | 50,567,276 | 55.07 |
| 4 | 94,647,122 | 8.84 | 190 | 85,571,480 | 53,287,111 | 62.27 |
| 5 | 115,836,226 | 16.69 | 179 | 94,234,656 | 56,821,236 | 60.30 |
| 6 | 112,076,146 | 13.19 | 195 | 96,483,491 | 57,312,920 | 59.04 |
| 7 | 53,373,768 | 9.74 | 192 | 47,760,676 | 29,565,735 | 61.90 |
| 8 | 56,971,994 | 4.09 | 189 | 53,814,265 | 31,934,946 | 59.34 |
| 9 | 127,304,662 | 7.31 | 184 | 116,836,341 | 69,392,637 | 59.39 |
| 10 | 67,098,716 | 10.97 | 256 | 59,131,644 | 32,329,948 | 54.67 |
| 11 | 94,968,416 | 16.54 | 191 | 78,472,585 | 47,698,776 | 60.78 |
| 12 | 75,320,750 | 6.97 | 184 | 68,829,138 | 34,266,942 | 49.79 |
| 13 | 104,423,616 | 9.73 | 188 | 93,340,333 | 56,604,488 | 60.64 |
| 14 | 99,823,954 | 9.31 | 197 | 89,778,012 | 52,210,422 | 58.16 |
| 15 | 91,317,458 | 8.06 | 197 | 82,971,980 | 49,646,633 | 59.84 |
| 16 | 73,776,006 | 12.63 | 192 | 63,637,858 | 36,378,240 | 57.16 |
| 17 | 82,108,918 | 6.66 | 185 | 75,351,532 | 44,109,773 | 58.54 |
| 18 | 103,799,282 | 10.90 | 186 | 86,421,364 | 47,295,490 | 54.73 |
| 19 | 72,155,772 | 4.88 | 192 | 67,784,241 | 39,993,189 | 59.00 |
| 20 | 92,006,314 | 12.56 | 182 | 79,405,782 | 48,127,323 | 60.61 |
| 21 | 98,195,572 | 11.34 | 189 | 86,263,893 | 51,857,578 | 60.12 |
| 22 | 93,614,060 | 10.43 | 176 | 82,121,935 | 48,157,258 | 58.64 |
| 23 | 101,864,758 | 7.37 | 183 | 93,180,126 | 57,396,611 | 61.60 |
| 24 | 107,413,162 | 7.52 | 200 | 98,484,603 | 59,123,749 | 60.03 |
| 25 | 93,800,710 | 10.71 | 188 | 82,858,619 | 51,706,402 | 62.40 |
| 26 | 111,187,298 | 12.61 | 195 | 96,454,720 | 59,408,762 | 61.59 |
| Mean | 92,445,842 | 9.82 | 192 | 81,940,405 | 48,312,011 | 58.84 |

*Following duplicate removal.

**Supplementary Table 3: The percentage of target nucleotides for each gene with a read depth** ≥**30**

| **Sample** | ***AHI1* (%)** | ***ARL13B* (%)** | ***B9D1* (%)** | ***B9D2* (%)** | ***C2CD3* (%)** | ***C5orf42* (%)** | ***CC2D2A* (%)** | ***CEP41* (%)** | ***CEP104* (%)** | ***CEP290* (%)** | ***CSPP1* (%)** | ***IFT172* (%)** | ***INPP5E* (%)** | ***KIAA0586* (%)** | ***KIF7* (%)** | ***KIF14* (%)** | ***MKS1* (%)** | ***NPHP1* (%)** | ***NPHP3* (%)** | ***OFD1* (%)** | ***PDE6D* (%)** | ***PIBF1* (%)** | ***RPGRIP1L* (%)** | ***TCTN1* (%)** | ***TCTN2* (%)** | ***TCTN3* (%)** | ***TMEM107* (%)** | ***TMEM138* (%)** | ***TMEM216* (%)** | ***TMEM231* (%)** | ***TMEM237* (%)** | ***TMEM67* (%)** | ***TTC21B* (%)** | ***ZNF423* (%)** | **Mean (%)** |
| --- | --- | --- | --- | --- | --- | --- | --- | --- | --- | --- | --- | --- | --- | --- | --- | --- | --- | --- | --- | --- | --- | --- | --- | --- | --- | --- | --- | --- | --- | --- | --- | --- | --- | --- | --- |
| 1 | 83 | 97 | 85 | 100 | 94 | 93 | 85 | 87 | 92 | 75 | 99 | 94 | 81 | 90 | 79 | 91 | 92 | 93 | 93 | 73 | 92 | 92 | 85 | 85 | 94 | 97 | 100 | 88 | 89 | 91 | 87 | 81 | 94 | 100 | 87 |
| 2 | 80 | 94 | 84 | 95 | 90 | 93 | 82 | 80 | 92 | 69 | 94 | 92 | 78 | 80 | 71 | 92 | 91 | 91 | 92 | 69 | 90 | 72 | 78 | 81 | 90 | 95 | 99 | 64 | 88 | 90 | 87 | 75 | 88 | 100 | 83 |
| 3 | 87 | 98 | 83 | 100 | 94 | 94 | 84 | 90 | 94 | 77 | 98 | 94 | 81 | 90 | 84 | 92 | 91 | 96 | 95 | 70 | 94 | 88 | 85 | 86 | 97 | 97 | 100 | 90 | 87 | 92 | 94 | 82 | 93 | 100 | 88 |
| 4 | 97 | 97 | 89 | 100 | 96 | 96 | 87 | 93 | 98 | 85 | 97 | 93 | 87 | 96 | 80 | 94 | 95 | 97 | 93 | 73 | 100 | 89 | 93 | 85 | 96 | 99 | 100 | 93 | 91 | 90 | 98 | 88 | 98 | 100 | 90 |
| 5 | 94 | 97 | 85 | 100 | 95 | 96 | 88 | 91 | 95 | 88 | 99 | 93 | 80 | 92 | 79 | 96 | 95 | 99 | 94 | 50 | 95 | 95 | 91 | 87 | 97 | 98 | 100 | 95 | 91 | 91 | 96 | 88 | 96 | 100 | 89 |
| 6 | 88 | 97 | 93 | 100 | 95 | 95 | 87 | 90 | 95 | 80 | 98 | 97 | 88 | 87 | 83 | 91 | 96 | 95 | 92 | 41 | 97 | 87 | 85 | 86 | 95 | 100 | 100 | 95 | 92 | 95 | 97 | 81 | 94 | 100 | 89 |
| 7 | 76 | 84 | 55 | 91 | 86 | 82 | 75 | 63 | 73 | 66 | 82 | 74 | 51 | 80 | 53 | 73 | 66 | 75 | 78 | 54 | 88 | 55 | 78 | 70 | 85 | 82 | 98 | 53 | 87 | 63 | 80 | 63 | 86 | 98 | 72 |
| 8 | 81 | 90 | 67 | 83 | 82 | 83 | 76 | 65 | 79 | 68 | 88 | 82 | 68 | 77 | 55 | 84 | 86 | 83 | 86 | 59 | 80 | 58 | 71 | 70 | 82 | 87 | 99 | 50 | 83 | 74 | 87 | 58 | 89 | 99 | 75 |
| 9 | 94 | 98 | 90 | 100 | 97 | 97 | 90 | 95 | 98 | 88 | 100 | 97 | 89 | 91 | 82 | 95 | 98 | 97 | 98 | 82 | 96 | 95 | 92 | 87 | 97 | 99 | 100 | 99 | 91 | 94 | 99 | 87 | 97 | 100 | 92 |
| 10 | 88 | 90 | 66 | 62 | 91 | 85 | 77 | 61 | 81 | 71 | 85 | 79 | 47 | 82 | 59 | 88 | 75 | 87 | 87 | 62 | 97 | 67 | 85 | 66 | 93 | 95 | 99 | 43 | 75 | 67 | 91 | 66 | 90 | 99 | 76 |
| 11 | 88 | 96 | 82 | 100 | 89 | 94 | 82 | 91 | 94 | 80 | 97 | 92 | 86 | 88 | 79 | 93 | 94 | 96 | 94 | 38 | 94 | 87 | 85 | 83 | 90 | 99 | 100 | 79 | 89 | 91 | 92 | 85 | 95 | 100 | 87 |
| 12 | 90 | 91 | 78 | 89 | 92 | 87 | 79 | 74 | 87 | 74 | 90 | 83 | 67 | 83 | 67 | 86 | 80 | 89 | 90 | 61 | 97 | 80 | 82 | 76 | 91 | 93 | 100 | 82 | 80 | 88 | 95 | 72 | 89 | 100 | 82 |
| 13 | 96 | 98 | 85 | 97 | 96 | 96 | 88 | 91 | 96 | 87 | 98 | 94 | 87 | 90 | 79 | 95 | 96 | 98 | 98 | 77 | 96 | 94 | 91 | 87 | 96 | 98 | 100 | 88 | 92 | 91 | 100 | 88 | 95 | 100 | 91 |
| 14 | 89 | 96 | 89 | 99 | 94 | 94 | 87 | 90 | 95 | 79 | 98 | 95 | 86 | 87 | 78 | 91 | 94 | 95 | 95 | 46 | 96 | 86 | 87 | 84 | 93 | 97 | 100 | 93 | 93 | 91 | 96 | 84 | 95 | 100 | 88 |
| 15 | 91 | 97 | 85 | 100 | 95 | 95 | 85 | 82 | 97 | 78 | 97 | 93 | 82 | 84 | 76 | 93 | 95 | 92 | 95 | 44 | 96 | 85 | 88 | 85 | 94 | 99 | 100 | 94 | 94 | 90 | 96 | 82 | 95 | 100 | 88 |
| 16 | 86 | 92 | 73 | 98 | 87 | 92 | 77 | 86 | 88 | 74 | 93 | 87 | 73 | 85 | 67 | 90 | 85 | 92 | 89 | 31 | 89 | 74 | 77 | 75 | 84 | 97 | 98 | 71 | 88 | 87 | 82 | 71 | 87 | 100 | 81 |
| 17 | 85 | 94 | 80 | 97 | 93 | 91 | 80 | 90 | 91 | 72 | 94 | 90 | 81 | 88 | 78 | 91 | 95 | 90 | 90 | 35 | 88 | 87 | 82 | 80 | 92 | 96 | 100 | 86 | 89 | 88 | 83 | 75 | 92 | 100 | 85 |
| 18 | 82 | 94 | 89 | 96 | 92 | 92 | 82 | 87 | 93 | 68 | 97 | 94 | 83 | 88 | 78 | 87 | 91 | 70 | 88 | 69 | 91 | 85 | 81 | 82 | 91 | 97 | 100 | 90 | 88 | 93 | 83 | 71 | 91 | 100 | 85 |
| 19 | 84 | 93 | 82 | 91 | 90 | 91 | 78 | 87 | 92 | 71 | 94 | 90 | 74 | 84 | 68 | 90 | 91 | 90 | 85 | 62 | 90 | 76 | 77 | 79 | 88 | 96 | 100 | 81 | 82 | 85 | 81 | 73 | 89 | 100 | 83 |
| 20 | 88 | 93 | 86 | 100 | 92 | 92 | 82 | 80 | 96 | 73 | 96 | 93 | 80 | 83 | 76 | 90 | 94 | 93 | 93 | 71 | 92 | 78 | 86 | 81 | 92 | 97 | 100 | 77 | 92 | 90 | 95 | 79 | 91 | 100 | 86 |
| 21 | 90 | 96 | 86 | 100 | 95 | 93 | 84 | 82 | 96 | 81 | 98 | 95 | 86 | 84 | 81 | 92 | 95 | 95 | 96 | 41 | 95 | 84 | 85 | 83 | 93 | 98 | 100 | 93 | 88 | 89 | 92 | 82 | 93 | 100 | 88 |
| 22 | 87 | 96 | 85 | 100 | 93 | 90 | 83 | 80 | 94 | 68 | 95 | 93 | 84 | 83 | 77 | 88 | 94 | 82 | 87 | 35 | 93 | 79 | 83 | 82 | 94 | 97 | 100 | 85 | 93 | 91 | 88 | 74 | 90 | 100 | 85 |
| 23 | 92 | 97 | 91 | 100 | 95 | 95 | 85 | 90 | 96 | 84 | 98 | 94 | 84 | 87 | 79 | 94 | 95 | 95 | 96 | 49 | 97 | 91 | 91 | 87 | 95 | 99 | 100 | 88 | 87 | 93 | 98 | 86 | 93 | 100 | 89 |
| 24 | 97 | 99 | 88 | 100 | 95 | 97 | 87 | 94 | 98 | 92 | 100 | 95 | 90 | 95 | 83 | 98 | 97 | 97 | 98 | 54 | 96 | 97 | 94 | 88 | 98 | 100 | 100 | 95 | 91 | 96 | 100 | 91 | 98 | 100 | 92 |
| 25 | 97 | 97 | 87 | 100 | 95 | 96 | 86 | 94 | 96 | 90 | 98 | 92 | 81 | 92 | 78 | 95 | 91 | 99 | 96 | 47 | 100 | 93 | 94 | 84 | 96 | 99 | 100 | 91 | 89 | 90 | 96 | 92 | 97 | 100 | 90 |
| 26 | 98 | 98 | 91 | 100 | 96 | 97 | 88 | 96 | 98 | 91 | 99 | 97 | 85 | 92 | 83 | 97 | 94 | 99 | 96 | 81 | 100 | 95 | 94 | 84 | 96 | 100 | 100 | 97 | 93 | 92 | 100 | 94 | 98 | 100 | 93 |
| Mean | 89 | 95 | 83 | 96 | 93 | 93 | 83 | 85 | 92 | 78 | 95 | 91 | 79 | 87 | 75 | 91 | 91 | 92 | 92 | 57 | 94 | 83 | 85 | 82 | 93 | 97 | 100 | 83 | 89 | 88 | 92 | 80 | 93 | 100 | 86 |

Target intervals comprise coding exon sequences plus 20 nt of each flanking intron.

**Supplementary Table 4: Summary variant counts following data processing using the SNV / small indel detection pipeline**

| **Sample**  **number** | **Total variant count** | **Variants located in target genes** | **Variants located in target genes with a minor allele frequency*** ≤**0.05** |
| --- | --- | --- | --- |
| 1 | 33,219 | 71 | 4 |
| 2 | 33,476 | 66 | 5 |
| 3 | 34,350 | 74 | 9 |
| 4 | 37,446 | 77 | 11 |
| 5 | 34,952 | 79 | 7 |
| 6 | 33,534 | 68 | 11 |
| 7 | 31,789 | 59 | 7 |
| 8 | 33,077 | 65 | 3 |
| 9 | 32,926 | 73 | 11 |
| 10 | 33,323 | 59 | 10 |
| 11 | 34,052 | 66 | 9 |
| 12 | 32,980 | 72 | 11 |
| 13 | 32,872 | 69 | 4 |
| 14 | 33,693 | 72 | 8 |
| 15 | 33,302 | 85 | 13 |
| 16 | 32,669 | 81 | 7 |
| 17 | 33,679 | 59 | 3 |
| 18 | 33,413 | 66 | 11 |
| 19 | 32,901 | 65 | 7 |
| 20 | 33,278 | 59 | 8 |
| 21 | 34,096 | 75 | 10 |
| 22 | 38,486 | 80 | 9 |
| 23 | 33,206 | 64 | 7 |
| 24 | 34,252 | 76 | 16 |
| 25 | 33,514 | 67 | 5 |
| 26 | 33,442 | 69 | 13 |
| Mean | 33,766 | 70 | 8 |

*Queried databases include dbSNP and the NHLBI GO Exome Sequencing Project.
