# Supplementary figures and images for: Enhanced diagnostic yield in Meckel-Gruber and Joubert syndrome through exome sequencing supplemented with split-read mapping
Source: BMC Med Genet. 2016 Jan 4;17:1. doi: 10.1186/s12881-015-0265-z (PMC4700600; doi:10.1186/s12881-015-0265-z)

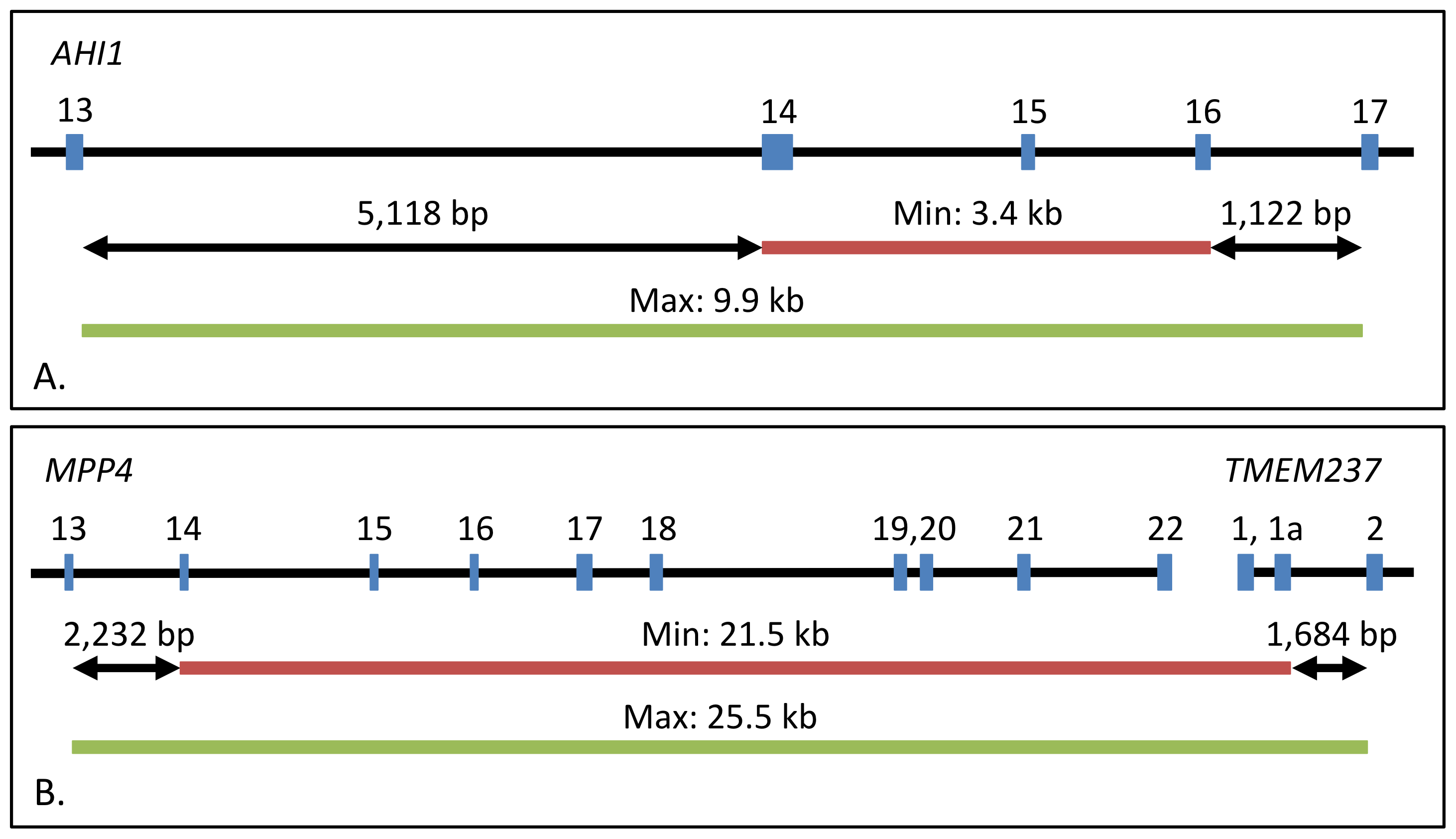

Supplement: Additional file 2: Figure S1. — Schematic representation of the FishingCNV-defined deletions showing the minimum (red) and maximum (green) possible boundaries of the deletion breakpoints for (A) the intragenic AHI1 deletion and (B) the TMEM237 to MPP4 deletion. Exons are displayed in blue and numbering is in accordance with transcripts [GenBank:NM_001134830.1] (AHI1), [GenBank:NM_001044385.2] and [GenBank:NM_152388.3] (TMEM237) and [GenBank:NM_033066.2] (MPP4). (TIF 19237 kb) [file 12881_2015_265_MOESM2_ESM.tif]
